# Cocaine-Induced Toxic Leukoencephalopathy, Serotonin Syndrome, and Central Diabetes Insipidus Following Cocaine Overdose: A Case Report of a Fatal Outcome

**DOI:** 10.7759/cureus.114922

**Published:** 2026-08-21

**Authors:** Ravina Kumari, Rommel Ramesh, Mathews P Thomas, Paria Sheibanimoghadam, Abdelnassir Abdelgabar

**Affiliations:** 1 Acute Medicine, Diana, Princess of Wales Hospital, Grimsby, GBR; 2 Family Medicine, South Georgia Medical Center, Valdosta, USA; 3 General Internal Medicine, Diana, Princess of Wales Hospital, Grimsby, GBR

**Keywords:** central diabetes insipidus, cocaine overdose, myonecrosis, serotonin syndrome, toxic leukoencephalopathy

## Abstract

Cocaine use is associated with a broad spectrum of neurological and systemic complications. Toxic leukoencephalopathy is a rare but severe manifestation characterized by diffuse white matter injury. Serotonin syndrome is a toxidrome resulting from excessive serotonergic activity. Central diabetes insipidus has rarely been described in association with toxic leukoencephalopathy and may indicate extensive hypothalamic or neuroendocrine injury.

We report a fatal case of a 44-year-old man presenting with toxic leukoencephalopathy, serotonin syndrome, and central diabetes insipidus following a massive cocaine overdose. Despite aggressive supportive management in the ICU, the patient developed progressive neurological deterioration and myonecrosis of his right upper arm, complicated by a radial artery pseudoaneurysm, in addition to his toxic leukoencephalopathy, serotonin syndrome, and central diabetes insipidus.

To the best of our knowledge, this is the first reported case describing the coexistence of toxic leukoencephalopathy, serotonin syndrome, central diabetes insipidus, and myonecrosis following a single massive cocaine overdose.

## Introduction

Cocaine remains one of the most frequently implicated illicit substances in drug-related emergency department (ED) presentations and is associated with substantial cardiovascular, neurological, and systemic morbidity [1,2]. Its primary pharmacological action involves the inhibition of presynaptic dopamine, norepinephrine, and serotonin reuptake, resulting in an excessive accumulation of synaptic catecholamines and monoamines [2,3]. Neurological complications of cocaine use include seizures, intracerebral hemorrhage, ischemic stroke, posterior reversible encephalopathy syndrome, and toxic leukoencephalopathy [3-5]. Diffuse toxic leukoencephalopathy is an uncommon but potentially devastating condition characterized by widespread white matter injury and severe neurological impairment [5,6].

Central diabetes insipidus following cocaine exposure has rarely been described. Proposed mechanisms include hypothalamic-pituitary ischemia resulting from profound vasoconstriction, vascular injury, or diffuse cerebral damage [7,8]. Furthermore, there are several published case reports describing cocaine-induced necrosis, including soft tissue necrosis, myonecrosis, and necrotizing fasciitis-mimicking presentations. This necrosis can become extensive enough to require repeated surgical debridement and/or amputation. The mechanisms involved include cocaine-induced vasoconstriction, ischemia, levamisole-induced immune vasculitis, severe agitation, hyperthermia, seizures, and prolonged immobilization. All of these factors can lead to rhabdomyolysis, compartment syndrome, and skeletal muscle necrosis [9,10].

## Case presentation

A 44-year-old man with a medical history of depression and anxiety was brought to the ED by ambulance after being found unconscious during a police welfare check. Collateral history from friends revealed the ingestion of approximately four bags of cocaine, with the patient reportedly unreachable for several days prior to discovery. Upon arrival, the patient was critically unwell; initial neurological assessment revealed a Glasgow Coma Scale (GCS) of 3/15 (E1, V1, M2) with decerebrate posturing. A generalized tonic-clonic seizure was witnessed shortly after his arrival in the ED. Initial physiological observations demonstrated pyrexia (38.2°C), hypoxia (oxygen saturation of 85% on room air), and hypotension (blood pressure of 101/57 mmHg).

Laboratory investigations demonstrated acute kidney injury and marked rhabdomyolysis (Table 1). A urine toxicology screen was positive for both benzoylecgonine and morphine, and chest radiography revealed bilateral infiltrates consistent with aspiration pneumonia. The patient was intubated for airway protection and admitted to the ICU. On day four of his admission, the patient developed persistent hyperthermia (>38°C), generalized rigidity, myoclonic jerks, and diffusely brisk reflexes. Following consultation with the National Poisons Information Service (TOXBASE), the patient's clinical features fulfilled diagnostic criteria for serotonin toxicity, although overlap with severe sympathomimetic toxicity was also considered. He was treated with intravenous midazolam and chlorpromazine, resulting in partial temperature control without significant neurological improvement.

**Table 1:** Initial laboratory investigations

Investigation	Value	Reference range
Creatinine	222 µmol/L	59–104 µmol/L
Creatine kinase	19,708 U/L	42-320 U/L
Sodium	136 mmol/L	133–146 mmol/L
Potassium	6.5 mmol/L	3.5–5.3 mmol/L
C-reactive protein	85 mg/L	0–5 mg/L
Estimated glomerular filtration rate	31 mL/min	90–200 mL/min
Alanine aminotransferase	590 U/L	0–41 U/L

**Figure 1:**
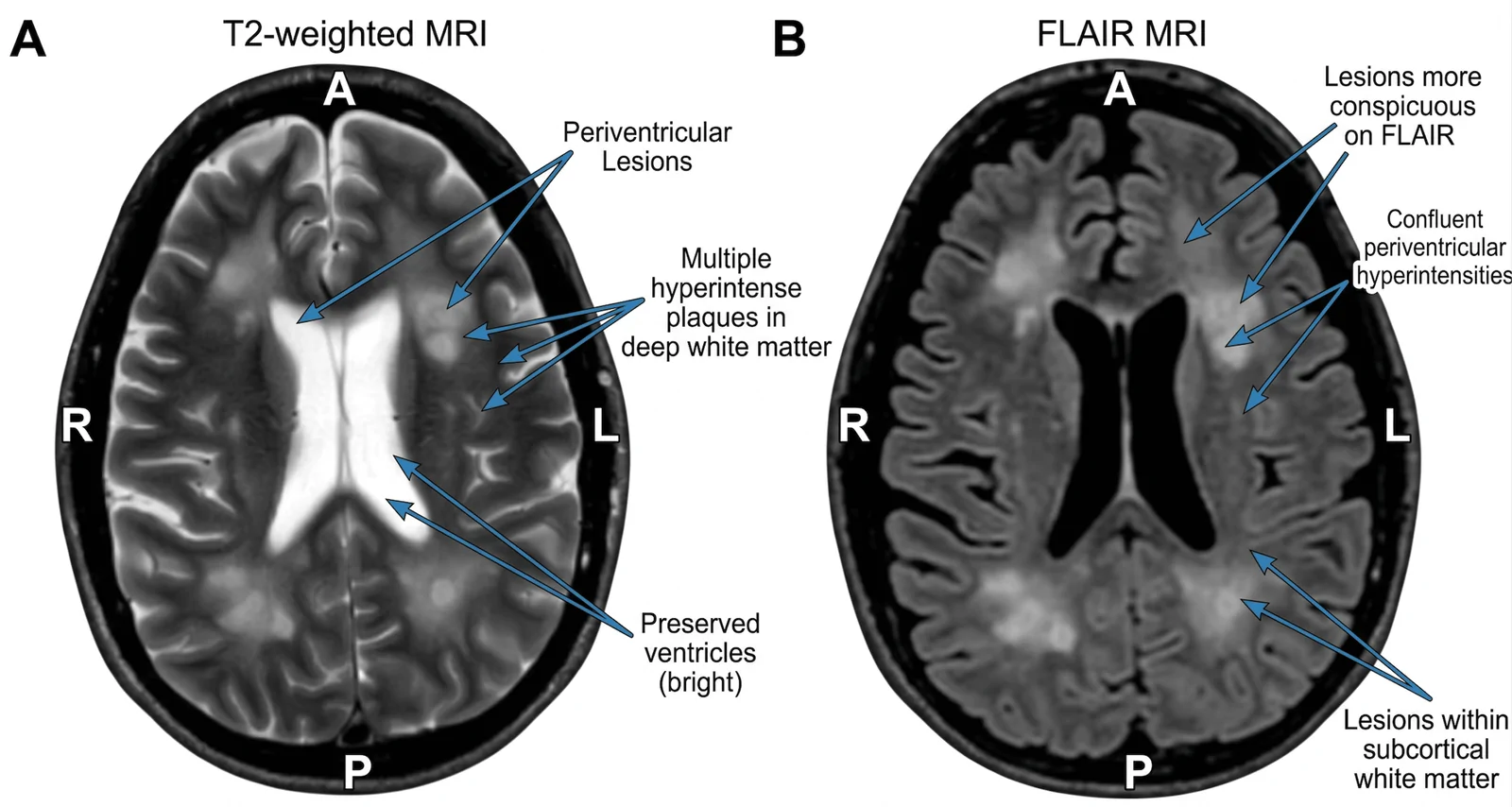
Axial T2-weighted (Panel A) and axial FLAIR (Panel B) MRI sequences demonstrate confluent, symmetrical periventricular, deep, and subcortical white matter hyperintensities consistent with diffuse toxic leukoencephalopathy. A: anterior; P: posterior; R: right; L: left; FLAIR: fluid-attenuated inversion recovery

An MRI of the brain demonstrated extensive bilateral, ill-defined, symmetrical T2 fluid-attenuated inversion recovery (FLAIR) hyperintensity involving the subcortical white matter, centrum semiovale, and corona radiata, extending to the margins of the lateral ventricles (Figure 1). These findings were consistent with diffuse toxic leukoencephalopathy, alongside focal lesions suggestive of small-vessel inflammatory or ischemic injury. An electroencephalogram (EEG) revealed a diffusely slowed, non-reactive background consistent with severe global encephalopathy.

To exclude alternative causes of his severe encephalopathy, a lumbar puncture was performed. Cerebrospinal fluid analysis demonstrated clear fluid with normal protein, glucose, and lactate dehydrogenase levels, with no leukocytes identified. An extensive autoimmune encephalitis screen was also undertaken, including antibodies against gamma-aminobutyric acid (GABA) receptors, voltage-gated potassium channel (VGKC) complex, contactin-associated protein-like 2 (CASPR2), leucine-rich glioma-inactivated 1 (LGI1), and N-methyl-D-aspartate receptor (NMDAR), all of which were negative. A vasculitis screen was similarly unremarkable, providing no evidence of an underlying autoimmune or inflammatory process.

During his prolonged ICU stay, the patient developed necrotizing fasciitis of the right upper limb, which was further complicated by a pseudoaneurysm of the right radial artery. He underwent urgent surgical debridement and arterial ligation, and subsequent blood cultures grew *Staphylococcus aureus*, requiring an extended course of intravenous antimicrobial therapy. Approximately three weeks into his admission, the patient developed profound polyuria with worsening hypernatremia (serum sodium peaking at 165 mmol/L). Urine studies supported a diagnosis of central diabetes insipidus (Table 2), and treatment with subcutaneous desmopressin (1 microgram once daily) was initiated, achieving a gradual normalization of his serum sodium levels.

**Table 2:** Diabetes insipidus workup

Investigation	Value	Reference range
Peak sodium	165 mmol/L	133–146 mmol/L
Serum osmolality	361 mOsm/kg	275–295 mOsm/kg
Urine output (24 hours)	4100 ml	800-2000 ml

Despite aggressive multidisciplinary management involving intensive care, neurology, endocrinology, microbiology, and palliative care teams, the patient's neurological condition remained static. Following discussions with his family, care was redirected toward comfort measures, and the patient died 38 days after admission.

## Discussion

Diffuse toxic leukoencephalopathy is a rare neurological complication associated with cocaine exposure and has been reported following both acute and chronic use [5,6,11]. Characteristic MRI findings include symmetrical T2-weighted and FLAIR hyperintensities involving the cerebral white matter, frequently accompanied by severe encephalopathy [5,6]. Proposed mechanisms include cerebral vasoconstriction, endothelial dysfunction, mitochondrial injury, oxidative stress, and direct oligodendrocyte toxicity leading to demyelination and white matter injury [4-6]. Although levamisole-adulterated cocaine has been implicated in inflammatory leukoencephalopathy, similar radiographic findings have been described in patients without evidence of adulterant exposure, supporting a direct neurotoxic effect of cocaine itself [11,12]. The radiological differential diagnosis includes hypoxic-ischemic injury, posterior reversible encephalopathy syndrome, toxic-metabolic encephalopathy, and inflammatory demyelinating disorders [4,5]. In the present case, the combination of extensive symmetrical white matter abnormalities and documented cocaine toxicity strongly favored toxic leukoencephalopathy.

Cocaine inhibits serotonin reuptake and may contribute to serotonin toxicity, particularly in the setting of massive ingestions or concomitant use of serotonergic medications [13,14]. Several reports have described serotonin syndrome associated with cocaine exposure, presenting with hyperthermia, rigidity, myoclonus, hyperreflexia, and autonomic instability [13-15]. The clinical features observed in our patient were consistent with a serotonin toxicity phenotype, although an overlap with severe sympathomimetic toxicity must be acknowledged.

The most unusual aspect of this case was the development of central diabetes insipidus. Cocaine-associated central diabetes insipidus has been reported only rarely in the literature [7,8]. Proposed mechanisms include ischemic injury to the hypothalamic-neurohypophyseal axis resulting from intense vasoconstriction, vascular thrombosis, or diffuse cerebral injury [7,8,16]. The marked hypernatremia, profound polyuria, biochemical findings, and the patient's response to vasopressin in this case strongly supported the diagnosis. Finally, the muscle necrosis, complicated by a radial artery pseudoaneurysm and staphylococcal septicemia, adds to the extreme and serious complications that can co-exist in the setting of a massive cocaine overdose [17,18].

## Conclusions

This case highlights the potential for severe cocaine toxicity to produce catastrophic multi-organ dysfunction extending far beyond classical cardiovascular and neurological complications. Diffuse toxic leukoencephalopathy should be heavily considered in patients with persistent encephalopathy following stimulant exposure, particularly when MRI demonstrates symmetrical white matter abnormalities. Furthermore, atypical serotonin syndrome may occur in massive cocaine overdoses, even in the absence of classical serotonergic medication exposure. The development of central diabetes insipidus is a rare but important complication of severe cocaine toxicity and may reflect extensive ischemic injury involving the hypothalamic-pituitary regulatory pathways; it should be suspected in the presence of unexplained hypernatremia and polyuria. Early recognition of these mixed toxidromes and secondary endocrine failures is essential, as these features are highly associated with a poor neurological prognosis, and their identification can assist in prognostication and guide multidisciplinary management.
